# Physiological Mechanisms of Exercise and Its Effects on Postural Sway: Does Sport Make a Difference?

**DOI:** 10.3389/fphys.2022.792875

**Published:** 2022-02-14

**Authors:** Erika Zemková

**Affiliations:** ^1^Department of Biological and Medical Sciences, Faculty of Physical Education and Sport, Comenius University in Bratislava, Bratislava, Slovakia; ^2^Sports Technology Institute, Faculty of Electrical Engineering and Information Technology, Slovak University of Technology, Bratislava, Slovakia; ^3^Faculty of Health Sciences, University of Ss. Cyril and Methodius in Trnava, Trnava, Slovakia

**Keywords:** athletes, general exercise, physiological mechanisms, postural stability, sport-specific exercise

## Abstract

While the effect of a variety of exercises on postural balance control has been extensively studied, less attention has been paid to those requiring sport-specific skills. Therefore there is a need to analyze the literature and elucidate changes in postural balance control after exercises performed in conditions close to a particular sport. This scoping review aims (i) To map the literature that addresses postural sway aspects of acute responses to general and sport-specific exercises and their underlying physiological mechanisms, and (ii) To identify gaps in the existing literature and propose future research on this topic. The main literature search conducted on MEDLINE, Web of Science, Scopus, PubMed, and Cochrane Library databases was completed by SpringerLink, Elsevier, and Google Scholar. A total of 60 articles met the inclusion criteria. Findings identified that among a variety of studies evaluating the effects of exercise on postural balance control, only few of them were conducted under sport-specific conditions (i.e., while shooting in biathlon or pentathlon, and after simulated or match-induced protocols in combat and team sports). Therefore, more research is still needed to address this gap in the literature and aim research at investigation of postural sway response to sport-specific exercises. Further analysis of the literature showed that the type, intensity and duration of exercise play a key role in increased postural sway. Whole body and localized muscular fatigue of the trunk, neck and lower limbs is considered to be a main factor responsible for the magnitude of balance impairment in an initial phase of recovery and speed of its readjustment to a pre-exercise level. Other likely factors affecting postural stability are hyperventilation and deterioration of sensorimotor functions, though some contribution of muscle damage, dehydration, hyperthermia or dizziness cannot be excluded. A better understanding of the physiological mechanisms of balance impairment after exercises performed under simulated fatigue induced protocol, close to conditions specific to a particular sport, has implications for designing smart exercise programs tailored to individual needs to improve athlete performance with high demands on postural stability and/or decrease their risk of injuries.

## Introduction

Balance is one of the limiting factors of performance in many sports. While static balance is important in shooting or archery, in free style sports, snowboarding, skateboarding, windsurfing or cycle acrobacy, dynamic balance plays an essential role in performance. In some sports, like gymnastics, ballet, aerobics, yoga, tai-chi or karate, athletes are required to maintain balance in various sport-specific positions. Others are sports where biomechanical stability for maintenance of balance is limited by a narrow area of support, such as figure-skating, ice-hockey, climbing or mountaineering. Other representative examples are canoeing, rowing, and equestrian sports where balance control in a sitting position is required. Control of body position during and after sport-specific exercises (e.g., body rotations) is necessary in dancing and ballet. Similarly, performance in gymnastics, figure-skating or rock and roll dancing is based on precise regulation of the center of mass movements. This ability also plays some role in weightlifting, powerlifting, golf and throwing events. This also includes most combat sports like boxing, fencing, karate, tae-kwon-do, wrestling and judo. The loss of balance in these injury-prone activities, including martial arts, may not only affect athlete performance but also increase the risk of injuries. Likewise, impairment of postural stability by high vertical forces produced during intensive bouncing exercises in acrobatic sports may cause back pain or lower limb injuries (e.g., ankle sprains). Loosing balance while performing side-to-side movements in individual and team sports, for example badminton, basketball, handball, field-hockey, soccer, softball, squash, table tennis, tennis or volleyball, may also contribute to lower limb injuries (e.g., anterior cruciate ligament sprain or tear). Last but not at least are long-term events such as biathlon, running, cycling, track and field and cross-country skiing, or sports requiring specific technical skills like hurdling and skiing, after which an increased postural sway may be observed when compared to baseline.

Such an exercise induced balance impairment does not only affect the outcome, but may also increase the risk of injuries (Zemková, 2014). Therefore, rapid readjustment of balance after sport-specific exercise to baseline is considered to be an important ability. Acute and chronic adaptations in postural control in response to different forms of exercise have been extensively researched and found to be beneficial for designing sport-specific and rehabilitation programs for balance improvements. Postural sway response to exercise was found to depend on its type, intensity, duration, intensity of proprioceptive stimulation, forms of muscle contraction and activation of muscle fibers (Zemková and Hamar, 2014). The review by Paillard (2012) revealed that short and intensive general exercise (involving the whole body) increases postural sway when the energy expenditure exceeds the lactate accumulation threshold. Hyperventilation rather than fatigue is responsible for increased postural sway after short-term intensive exercises (Zemková and Hamar, 2014). Exhaustive local exercise (involving a particular muscular group) affects postural control when it generates a strength loss at least 25–30% of maximal voluntary contraction (Paillard, 2012). Non-intensive general and local exercises can also disturb postural control when the exercise is prolonged (Paillard, 2012). Fatigue is usually considered as a main factor responsible for balance impairment after prolonged exercise (Zemková and Hamar, 2014). Both general and local exercises contribute to altering the effectiveness of sensory inputs and motor output of postural control (Paillard, 2012). Thus, impairments of sensorimotor functions very likely also play a role in increased post-exercise postural sway (Zemková and Hamar, 2014). This assumption may be corroborated by significant differences in balance impairment after exercises that induced the same ventilation but with a different intensity of muscle contractions eliciting a different level of proprioceptive stimulation, such as calf raises versus jumps and cycling versus running (Zemková and Hamar, 2014). Different compensatory postural strategies are triggered to counteract or limit the disturbance of postural control due to general and local muscle fatigue (Paillard, 2012).

Particularly highly skilled athletes are able to perform successfully in spite of increased postural sway. For instance, gymnasts and ice-hockey players while standing on a narrow area of support, mountaineers during a stance at a height of about 20 m above the ground, skiers and snowboarders on an unstable surface with fixed ankle joints, weightlifters and bodybuilders when performing barbell squats with an additional load, shooters during repeated shots or basketball players during repetitive free throw shots (Zemková, 2014).

Practicing any kind of sport is associated with improved postural stability (Andreeva et al., 2021). Postural adaptations occur in trained subjects because elite athletes exhibit better postural performance and different postural strategy than sub-elite athletes (Paillard, 2017). Adaptations are specific to the context in which the physical activity is practiced (Paillard, 2017). The most successful athletes in terms of sport competition level have the best postural performance both in ecological (specific postural conditions related to the sport practiced) and non-ecological (decontextualized postural conditions in relation to the sport practiced) postural conditions (Paillard, 2019). They also have more elaborate postural strategies compared with athletes at lower competition level (Paillard, 2019).

However, research to date has only marginally addressed the acute effect of sport-specific exercises on postural balance control and their underlying physiological mechanisms. Given the absence of information in a number of such studies, their design and findings, reviews in this field of balance research are warranted. There is a clear need for analysis of the existing literature to elucidate the effects of sport-specific exercises on postural balance control in athletes. A better understanding of physiological changes induced by exercises performed in specific conditions of a particular sport would provide the basis for designing smart balance training programs tailored to individual athletes. This scoping review aims (i) To map the literature that addresses postural sway aspects of acute responses to general and sport-specific exercises and their underlying physiological mechanisms and (ii) To identify gaps in the existing literature and propose future research on this topic.

## Methods

The paper was designed as a scoping review (Armstrong et al., 2011; Sucharew and Macaluso, 2019). Two specific questions were addressed in this review: (1) Does sport specialization play a role in postural sway response to exercise? and (2) What are physiological mechanisms underlying acute effects of exercise on postural sway?

An electronic literature search was provided to analyze existing studies dealing with acute changes in postural balance control induced by various exercises, particularly sport-specific exercises, and their underlying physiological mechanisms. Studies were searched on Web of Science, SCOPUS, PubMed, MEDLINE, and Cochrane Library databases. This search was completed on Google Scholar, SpringerLink, and Elsevier. The articles in peer-reviewed journals were considered for analysis. A manual search for references included in reviews was also conducted to identify further relevant studies. If multiple papers included overlapping data resulting from the same or similar studies, the one with the most recent publication date was analyzed. Articles or abstracts published in conference proceedings, theses, case studies and books were excluded. Articles were also excluded if they did not contain original research or were incomplete. The inclusion criteria involved research articles that specified participants, experimental protocols and measures relevant to this review. The literature search was limited to English language. Articles published after 1990 were preferred, however, earlier relevant studies were also included. Articles that failed to meet the eligibility criteria for this review were excluded.

The initial search was confined to research articles related to the main purpose of this review, i.e., those investigating the effects of sport-specific exercises on postural balance control. Thus, the key inclusion criterion was exercises performed under conditions specific to a particular sport. However, this approach revealed only a limited number of papers that met the eligibility criteria. The search was therefore widened to investigations dealing with acute changes in postural balance control after exercises of different type, intensity, and duration. In particular, physiological mechanisms underlying these changes were studied. These together helped to identify gaps in the existing literature and formulate recommendations for future research on this topic.

The search and appraisal of selected studies on the basis of exclusion and inclusion criteria was performed by the author of this review. Some concerns were related to sample representativeness, missing information about the control group and/or non-controlled compliance of experiments. The target population was athletes of team and individual sports where balance can play a role in their performance, as described in the first paragraph of this review. Proposed sports were combined with the following keywords.

**FIGURE 1:**
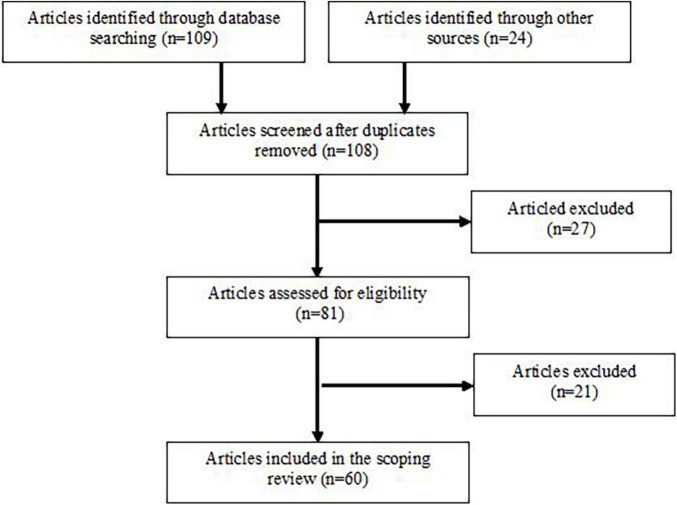
Flow chart illustrating phases of the literature search and study selection.

A combination of these terms was included in the search strategy: “acute effect ‘AND’ post-exercise response ‘AND’ balance ‘AND’ postural control ‘AND’ athletes ‘AND’ sport-specific exercise ‘AND’ type of exercise ‘AND’ exercise intensity ‘AND’ duration of exercise ‘AND’ physiological mechanisms.” Further searches were conducted using words from subheadings that for example, specified the form of exercise in a particular sport or postural sway variables used. Altogether 133 articles were identified through database searching. Following an initial screening and assessing for eligibility, articles that failed to meet the inclusion criteria were removed. 60 articles that investigated the effect of general and sport-specific exercises on postural balance control were included in this scoping review. Particular phases of the search process are shown in Figure 1.

## Results and Discussion

### Acute Postural Sway Response to Exercises and Their Underlying Physiological Mechanisms

First of all, studies investigating postural sway response to sports-specific exercises were analyzed. The next step was to analyze studies that involved the effects of a variety of exercises on postural balance control while attention was paid to those discussing potential physiological mechanisms underlying these changes. This search included 60 studies (Supplementary Table 1).

Evidence based on the analysis of center of pressure (CoP) sway area and/or velocity velocity in 936 athletes of 13 groups of sports, such as (i) basketball, handball, (ii) biathlon, practical shooting, archery, trapshooting, (iii) boxing, karate, kickboxing, thai boxing, taekwondo, (iv) tennis, table tennis, (v) alpine skiing, snowboarding, (vi) figure skating, freestyle, (vii) football, (viii) rowing, canoeing, canoe slalom, (ix) freestyle wrestling, Greco-Roman wrestling, judo, sumo, (x) speed skating, curling, hockey, short track, (xi) cross-country skiing, (xii) sprint running, stayer running, orienteering, (xiii) artistic gymnastics, rhythmic gymnastics, cheerleading, trampoline tumbling, climbing, high jumping, sailing, skeleton luge, showed that their postural stability is better than in the non-athlete control group (Andreeva et al., 2021).

Good postural stability in shooters results from assiduous training aimed at improving balance (Aalto et al., 1990). They have significantly lower sway velocity compared to untrained subjects both with and without the competition clothing (Aalto et al., 1990). Higher Romberg quotient in shooters than in controls indicates that they use an increased amount of vestibular and proprioceptive cues to stabilize their posture (Aalto et al., 1990). Top-level rifle shooters can stabilize their posture during aiming before the shot better than naive shooters (Era et al., 1996). However, their worse posture stabilization during the last seconds preceding the shot seems not to be a reason for poor shooting performance (Era et al., 1996). Investigating shooting accuracy and precision for prone shooting in biathletes revealed that the exercise intensity has minimal influence on prone shooting performance, but does affect shooting in the standing position by altering the stability of the hold (Hoffman et al., 1992). Maximal physical effort performed on a ski ergometer until exhaustion influences both postural and rifle stability during aiming in biathletes (Sadowska et al., 2019a). Similarly, the running effort within the Laser Run affects the stability of the shooting position in pentathletes (Sadowska et al., 2019b). The main factor hindering accurate and fast shooting is fatigue (Sadowska et al., 2019b). However, the fatigue level does not affect the magnitude of the disturbances of postural balance in the shooting position (Sadowska et al., 2019b).

Postural stability and its readjustment after sport-specific exercises is also important in acrobatic, combat and collision-based team sports. Training consisting of balance exercises usually contributes to better postural stability in highly skilled athletes compared to those at lower level of competition or untrained individuals. For instance, expert gymnasts and experts in other non-gymnastic sports demonstrate larger postural sway in the unipedal tasks when vision is removed; however, this effect is less accentuated for the gymnasts (Vuillerme et al., 2001). Gymnasts are able to use the remaining sensory modalities to compensate for the lack of vision in unstable postures (Vuillerme et al., 2001).

Further, linear and angular sway velocity are lower in a squat position but not in a bipedal stance in wrestlers compared to controls (Mel’nikov et al., 2012). Fatigue induced by PWC (170) test on the cycle ergometer increases postural sway in both bipedal stance and squat position (Mel’nikov et al., 2012). While the linear sway velocity in bipedal stance after PWC (170) increases to an equal extent in both groups, in a squat position it is lower in athletes (Mel’nikov et al., 2012).

In team sports, sensorimotor impairments resulting from previous injuries or muscular fatigue can be a factors contributing to an increased injury risk (Zech et al., 2012). For instance, higher sway area in basketball players than in controls can be related to their history of ankle trauma (Perrin et al., 1991). However, static postural sway measures may be insufficient to allow conclusive statements regarding sensorimotor control in the non-injured athletes (Zech et al., 2012). The CoP sway velocity during a single-leg stance on a force plate increases after both treadmill running and single-leg step-up exercises in handball players, whereas there is no fatigue effects for the star excursion balance test (Zech et al., 2012). Although fatigue affects static postural control, sensorimotor mechanisms responsible for regaining dynamic balance in healthy athletes seem to remain predominantly intact (Zech et al., 2012). On the other hand, soccer match-induced fatigue increases drop jump ground contact time concomitant with the impairment of dynamic balance and agility performance when moving short distances, whereas there are no significant changes in agility performance on longer movement distances, explosive power of lower limbs, static balance, speed of step initiation and the soccer kick (Zemková and Hamar, 2009b). Another study showed that the area of the CoP trajectory during a postural task is larger after a Canadian football G-Sim along with reductions in peak isometric knee extensor torque, peak power and take-off velocity during a countermovement jump (Clarke et al., 2015). These changes may be ascribed to alterations in excitation-contraction coupling due to structural damage and central activation failure (Clarke et al., 2015). The authors suggest that both neuromuscular and somatosensory alterations are induced by acute game-induced fatigue in collision-based team sports players.

As a consequence of match-induced fatigue may be an increased risk of injuries. There is a high correlation (0.92) between delays in peroneal muscle reaction time (onset of EMG following sudden ankle inversion) and increases in postural sway amplitude (Konradsen and Ravn, 1991). This delay in peroneal reaction time results in a delay of muscle force generation, which is similar to the electromechanical delay of muscle force generation seen with muscular fatigue (Häkkinen and Komi, 1983; Hortobágyi et al., 1991). Delay in muscle force generation leads to an increase in unilateral postural sway amplitude and may result in lower limb injury during long-term athletic activity (Tropp et al., 1984). Thus, if the forces required for the correction of an unstable placement of the foot are delayed due to fatigue, then mainly ankle joints would be at risk of injury. Ankle sprains and rupture of the anterior cruciate ligament are among the most serious injuries in players. These injuries occur mostly in the end of the match, which raises the possibility that muscular fatigue at the ankle and knee joints would place players at greater risk of injury.

Most of the studies considered fatigue as a main factor responsible for post-exercise balance impairment. The increase in postural sway was revealed after fatigue of ankle plantar-flexor muscles (Gimmon et al., 2011) induced by isokinetic contractions (Lundin et al., 1993; Yaggie and McGregor, 2002), isometric and isokinetic exercises (Bisson et al., 2012), repeated plantar-flexion of both legs (Corbeil et al., 2003), toe-lift until exhaustion (Pinsault and Vuillerme, 2008), single-leg, weight-bearing heel raises on an inclined platform (Springer and Pincivero, 2009), repeated standing heel raise exercise (Hlavackova and Vuillerme, 2012), and a calf raise exercise on top of a step (Barbieri et al., 2019). Also fatigue induced by voluntary muscular contraction or electrical stimulation applied to the dorsi-plantar flexor (Yu et al., 2014), the triceps surae (Magalhães and Kohn, 2012), and the quadriceps femoris (Paillard et al., 2010; Chaubet et al., 2012; Chaubet and Paillard, 2012) can impair postural control. Furthermore, it is lower extremity joint (knee, or ankle) and overall fatigue of the dominant leg (Dickin and Doan, 2008), fatigue induced by a high-intensity free-weight back-squat exercise (Thiele et al., 2015), lumbar extensor fatigue (Davidson et al., 2004; Pline et al., 2005) induced by a trunk extension-flexion exercise on a roman chair (Parreira et al., 2013), cervical muscular fatigue (Vuillerme et al., 2005), and neck musculature fatigue (Liang et al., 2014). Moreover, whole-body fatigue in a form of exercise on a rowing ergometer (Springer and Pincivero, 2009), maximal and submaximal cycle ergometry (Hill et al., 2014), cycle ergometry and treadmill walking (Hill et al., 2015), 25 min of moderate running on a treadmill (Marcolin et al., 2019), Wingate test on a cycle ergometer (Wojciechowska-Maszkowska et al., 2012), two times of a maximal voluntary pedaling for 10 s and at 50% of maximal aerobic power for 60 min at 60 rpm (Demura and Uchiyama, 2009), and maximum-effort sprints and yo-yo intermittent recovery test, level 1 exercise (Fox et al., 2008) plays a role in increased values of postural sway. The impairment of a single-leg dynamic balance is higher after aerobic (the Bruce protocol on a motorized treadmill) than anaerobic exercise (four maximal cycling efforts against a resistance equivalent to 0.075 kg/body mass for 30 s with 3-min rest intervals) (Güler et al., 2020). While the overall stability index and the anterior/posterior index increases significantly immediately following the fatiguing treadmill test, their values are not altered significantly after an incremental test on a cycle ergometer (Wright et al., 2013). There are no significant differences in the equilibrium and strategy scores after maximal exercise on the cycle ergometer as compared to baseline, neither with eyes open nor with eyes closed while standing on a stable platform, however, these values measured under dynamic conditions are significantly lower than prior to exercise with eyes closed as well as with sway-referenced vision (Zemková et al., 2007). Sensory analysis reveals that the vestibular system is more affected by exercise than the somatosensory system.

Findings indicate that the effect of exercise on balance depends mainly on its type, intensity and duration. While strenuous physical exercise (treadmill walking and cycle ergometer pedaling) increases body sway, it is little affected by exercise performed below the estimated anaerobic threshold (Nardone et al., 1997). Postural sway is also affected by prolonged fatiguing exercise in a form of treadmill walking for 25 min (Nardone et al., 1998). In both cases this effect is of moderate extent and short-lasting (Nardone et al., 1997, 1998). It seems that abrupt intensive exercise has a more profound but shorter detrimental effect on balance than prolonged exercise of moderate intensity. While the impairment of balance in the first case is mediated mainly by hyperventilation, fatigue is responsible for longer balance disorders in the second case.

Therefore both the magnitude of balance impairment in an initial phase of recovery as well as the speed of its readjustment should be taken into account when evaluating postural sway response to exercise (Zemková and Hamar, 2014). Since the adjustment of sway velocity at the onset of post-exercise recovery phase seems to be exponential, it may be characterized by the time required to decline to 50% of the difference between maximal sway velocity (achieved in an initial 5-s post-exercise phase) and steady state balance (t_1/2_). This time is longer after maximal stepwise running and cycling than after abruptly instituted exercise. However, it may take few minutes to return postural sway to the pre-exercise level after prolonged or intensive exercises. One may identify three phases in the recovery period. In an initial phase of recovery there is a plateau or slight increase in the CoP velocity, which is most probably associated with a re-payment of ventilation. Second is a fast exponentional component in the decline of CoP velocity, with a half-time of about 1 min. This phase is most likely associated with a decline in ventilation, as it has been demonstrated by close correlation between the reduction of ventilation and CoP velocity in the recovery period (Zemková and Hamar, 2003). Then comes a more complex, slow component. A sustained increase in the sway velocity during the last phase may be attributed to the impairment of proprioceptive feedback mechanisms involved in balance control. However, the contribution of fatigue or the deterioration of other components of sensorimotor system cannot be excluded.

Overall, our previous research identified higher increase in postural sway, and in some cases also its slower return to a baseline, after short-term abruptly instituted intensive than longer stepwise exercise on the cycle ergometer, prolonged (45 min) than shorter (15 min) cycling at moderate intensity, rebound jumps than calf raises, treadmill running than cycling, upslope than level running on the treadmill, and cycling at higher (130/min) than lower (70/min) revolution rates (Zemková and Hamar, 2014).

On the other hand, some authors have reported no significant changes in postural sway variables after exercise. A recent study by Lyu et al. (2021) evidenced a general compensation in the central nervous system in response to the neuromuscular deficiencies induced by local fatiguing exercise and put forward the function of sensory recalibration in maintaining postural stability under fatigue conditions. For instance, repeated measurements for up to 10 min after a fatiguing calf-muscle exercise showed no increase of body sway, which indicates that postural control in quiet standing can be maintained by compensatory mechanisms activated during muscle fatigue (Adlerton and Moritz, 1996). The next study by Adlerton and Moritz (2001) also revealed that fatiguing exercise does not influence the CoP shift caused by vibration, thus indicating unchanged excitability of muscle spindles in fatigued muscles. Though calf-muscles fatigue does not impair postural control, it generates a change of the contribution of the proprioceptive information (myotatic loops), which is greater after voluntary muscular contractions than after electrical stimulation superimposed onto voluntary muscular contractions (Bizid et al., 2009). Similarly, ankle muscle fatigue does not affect postural variables under eyes open conditions, but with eyes closed sway area and antero-posterior velocity increases when both plantarflexors and dorsiflexors are fatigued simultaneously (Boyas et al., 2011). This may be ascribed to the impairment in the compensatory activity between agonist and antagonist muscles and/or a greater decrease in proprioception due to a greater number of fatigued muscles (Boyas et al., 2011). Fatiguing exercises consisting of sustaining plantarflexor isometric contractions at the intensity of 25, 50, and 75% of maximal isometric plantarflexor torque until task failure does not influence the extent of postural stability impairment, but does influence the type of fatigue induced and the neuromuscular function predictors explaining changes in postural variables (Boyas et al., 2013). Further, unilateral muscle fatigue induced on the hip’s abductors of the dominant leg yields to larger CoP displacements under the non-fatigued leg only (Vuillerme et al., 2009). This indicates that supplementary somatosensory inputs to the central nervous system preserves/facilitates postural control in the condition of altered neuromuscular function of the dominant leg’s hip abductors induced by the fatiguing exercise (Vuillerme et al., 2009). The other study by Strang and Berg (2007) showed that muscle fatigue generated by a dead-lift exercise performed to exhaustion has no effect on postural stability, and yet caused earlier anticipatory postural adjustment onsets in the contralateral paraspinals, ipsilateral paraspinals, and contralateral paraspinals.

These findings indicate that fatigue induced changes in the somatosensory system have to be taken into account when interpreting post-exercise changes in postural balance control. Prolonged exercise in a form of a 25-km run and ergocycle exercise of identical duration (on average 1 h 44 min) impairs postural stability during conflicting sensory conditions in well-trained triathletes, with some differences depending on the kind of exercise (Lepers et al., 1997). These athletes used vestibular inputs less effective after running than cycling while maintaining balance (Lepers et al., 1997). The authors suggest that adaptation to prolonged stimulation of proprioceptive, visual and vestibular inputs during exercise most likely occurred in the integrating centers. However, an impairment of motor efferents or hemodynamic changes can not be excluded. Also treadmill running at speed of 2.2 m/s tends to disturb postural stability more than walking at speed of 1.9 m/s, possibly due to more excessive head movement observed by larger vertical displacement and acceleration pattern, and disturbance of visual and vestibular information centers (Derave et al., 2002). As the study shown, exercise of moderate intensity increased two-dimensional postural sway in eyes open only. This deteriorated visual contribution to postural stability was evident as an initial destabilization in the sagittal direction and a less transient loss of latero-lateral stability (Derave et al., 2002). Likewise, Hashiba (1998) identified greater mean fore-back postural sway after treadmill running at a speed of 10 km/h than walking at a speed of 7 km/h. The author demonstrated that vision during treadmill locomotion plays an important role in evoking postural sway after such an exercise. Somatosensory/motor signals may be stored during visual-somatosensory/motor conflict and this stored information may evoke postural change and self-motion perception (Hashiba, 1998). Furthermore, prolonged (60 min) cycle ergometer exercise was found to increase the mean time interval between two consecutive peaks in sway density plot, thus decreasing the control rate but not changing the stability level (Mello et al., 2009). Conversely, the maximal oxygen uptake test caused a decrease of the mean duration of peaks in the sway density plot, decreasing the stability level, without modifying the rates of central and muscular torque controls (Mello et al., 2009). This means that visual privation has a more detrimental effect on body sway than these exercises, though it also depends on their intensity and duration (Mello et al., 2009). Fatiguing running also affects static and dynamic postural control in active athletes with previous ankle sprain (Steib et al., 2013). Their fatigue-induced alterations of dynamic postural control (Star Excursion Balance Test) were greater as compared to uninjured controls (Steib et al., 2013). This indicates that ongoing deficits in sensorimotor control may contribute to the enhanced ankle reinjury risk (Steib et al., 2013). Similarly, a latent impairment of balance performance was found following a bout of plyometric exercise consisting of 200 countermovement jumps designed to elicit symptoms of muscle damage (Twist et al., 2008). This has implications for both the use of skill-based activities and for increased injury risk following high-intensity plyometric training (Twist et al., 2008).

Another factor is dehydration. Prolonged exercise (cycled for 2 h at a power output equal to 57–63% VO_2_max) without fluid ingestion negatively affects postural stability, whereas there is no effect after exercise with fluid replacement (intake of 1.9l of a carbohydrate-electrolyte solution) or after thermal dehydration induced by seven 15 min consecutive sauna sessions (85°C, 50% rh) with no fluid replacement (Derave et al., 1998). However, more recent study by Mtibaa et al. (2018) demonstrated that hyperthermia impairs the proprioception and balance parameters due to heat-induced alterations in efferent and afferent signals to and from the muscle. The exercise that induced a mild dehydration, which increases proteinemia and leads to body mass loss, was found to impair balance in the standard situation and when the vestibular cue is reliable (Lion et al., 2010). There was a correlation between the decreased use of vestibular input and the dehydration level (Lion et al., 2010). Even though muscular fatigue could explain the decrease in postural performances, vestibular fluid modifications may also be involved by its influence on the intralabyrinthine homeostasis, thus lowering the contribution of vestibular information on balance control (Lion et al., 2010). Fatigue mainly alters muscular effectors and sensory inputs, such as proprioception, resulting in poor postural regulation (Gauchard et al., 2002). Fluid ingestion could be responsible for the preservation of muscular functions and of sensory afferences accurately regulating postural control (Gauchard et al., 2002).

Last but not at least, increased ventilation plays an important role in postural sway response to exercise (Zemková and Hamar, 2014). In general, body movements associated with paced respiration disturb the postural control system (Hunter and Kearney, 1981). The magnitude of the respiratory contribution to sway is constant over the normal range of respiratory rates and linearly relates to respiration amplitude (Hunter and Kearney, 1981). Interestingly, the respiratory component of the sway path is larger in seated than in standing subjects, indicating that sitting entails less instantaneous steadiness (Bouisset and Duchene, 1994). Moreover, the sway distance is greater when holding breath after inspiration than expiration, and increasing the respiration rate produces a greater postural sway (Jeong, 1991). Specifically, breath holding that leads to activation of postural control is more pronounced in athletes (Malakhov et al., 2014). The athletes’ postural system also compensates for hyperventilation more efficiently when compared to controls but with greater effort (Malakhov et al., 2014).

Investigation of postural sway after light (40 W), moderate (85 W), and heavy (125 W) work loads under conditions of wearing a full facepiece respirator but no respiratory protection device revealed that its values increase more quickly and in a more consistently linear fashion with increasing work load under the respirator than the non-respirator condition (Seliga et al., 1991). This may be attributed to the increasing work load-induced proprioceptive fatigue effect on the nervous system’s ability to process signals from proprioception systems incongruent with body sway (Seliga et al., 1991). Another study evaluated postural response to a strenuous treadmill exercise and a 3 s bilateral soleus muscle vibration after the strenuous exercise (Bove et al., 2007). There was a linear relationship between sway path and oxygen uptake, which indicates that body instability may be due to rapid recovery of oxygen uptake (Bove et al., 2007). However, the fatigue-induced body instability was not associated with postural sway response to soleus muscle vibration (Bove et al., 2007). Ventilatory demands regulate diaphragmatic force-generation during exercise, whereas diaphragmatic fatigue must be attributed to non-ventilatory controlled feedback mechanisms (Kabitz et al., 2008). Evidence clearly indicates that hyperventilation impairs postural stability (Malakhov et al., 2014). For instance, hyperventilation induced by a maximum-intensity, incremental cycling exercise is accompanied by an increase in postural sway, indicating a reduction in postural stability following a change in ventilatory drive (David et al., 2010, 2015). It appears that the compensation of respiratory disturbances for erect posture is less effective when minute ventilation increases (David et al., 2015). A close link between sway velocity and ventilation in an initial phase of recovery was also found after resistance exercises (Zemková and Hamar, 2009a). The highest increase in the CoP velocity as well as ventilation was found after squats, followed by calf raises, voluntary hyperventilation, biceps curls, and presses behind the neck (Zemková and Hamar, 2009a). Thus, voluntary hyperventilation also increases postural sway (Sakellari and Bronstein, 1997). This increase may be mediated by derangement of both peripheral and central somatosensory signals from the lower limbs (Sakellari et al., 1997). Hyperventilation disrupts mechanisms mediating vestibular compensation. It seems to spare vestibular reflex activity and cerebellar-mediated eye movements (Sakellari et al., 1997). All these factors have to be taken into consideration when interpreting physiological mechanisms underlying postural sway response to exercise.

### Gaps in Current Studies Investigating Acute Effects of Exercise on Postural Sway and Proposals for Future Research

Although there is a wide variety of studies investigating the effects of exercise on postural balance control, less attention has been paid to those performed under sport-specific conditions. Previous reviews revealed that an increase in post-exercise CoP velocity is lower and its readjustment to pre-exercise level is faster in experienced athletes, for instance after body rotations in dancers and synchronized swimmers or judo falls in judo competitors (Zemková, 2014). The magnitude of post-exercise balance impairment depends on the form of exercise simulating conditions of a particular sport (e.g., Latino American vs. rock and roll dancing), intensity (e.g., maximal vs. bouncing aerobic jumps), and their duration reflecting the duration of performance (Zemková et al., 2010). It seems that sensory functions are more profoundly affected by intensive jumps than motor functions of the task-oriented balance exercise based on visual feedback control of the center of mass (CoM) position (Zemková and Hamar, 2011). However, after reaching some level of deterioration of proprioceptive function, there is no further impairment of balance parameters.

In most studies, functional testing protocols have been usually used to evaluate the effect of exercise-induced fatigue on the postural control system. However, these laboratory experiments, including stepwise increasing exercise loads on a treadmill or cycle ergometer, in many ways represent artificial conditions. Nevertheless, these procedures provide standardized protocols and can also simulate the physiological demands of many sports. Despite the many advantages of laboratory diagnostics, such exercises do not reflect specific changes in the neuromuscular system induced by a particular sport. From both a practical and a theoretical point of view it is therefore equally important to investigate the effect of intermittent exercise on postural balance control, which better reflects the type of muscular activities encountered in most sports. The intermittent exercise at a high intensity level is an activity pattern, where periods of intense exertion are interspersed with periods of active or passive recovery. Therefore, simulated fatigue induced protocols should be used in order to be closer to specific conditions in a particular sport. Nonetheless, it seems that the intensity of exercise rather than its mode (e.g., continual vs. intermittent exercise) plays a role in an increased postural sway (Zemková and Dzurenková, 2009).

Based on the analysis of the literature, there are only few studies that evaluated the effects of sport-specific exercise on balance and how it may affect sport performance, for instance in biathletes (Hoffman et al., 1992; Sadowska et al., 2019a), pentathletes (Sadowska et al., 2019b), wrestlers (Mel’nikov et al., 2012), handball (Zech et al., 2012), soccer (Zemková and Hamar, 2009b), or football players (Clarke et al., 2015). Therefore, further studies should be focused on the investigation of changes in postural sway following exercises performed under sport-specific conditions.

Post-exercise balance impairment is often associated with fatigue induced by prolonged exercises. On the other hand, more marked ventilation is responsible for increased postural sway after short-term intensive exercises, though some contribution of fatigue cannot be excluded. This was demonstrated by the close correlation between sway velocity and ventilation in an initial phase of recovery after intensive cycling bouts (Zemková and Hamar, 2003) as well as after resistance exercises (Zemková and Hamar, 2009a). The contribution of respiration to postural sway is low during quiet breathing but linearly increases with the respiration amplitude (Hunter and Kearney, 1981; Hodges et al., 2002). Although a threshold for posture-destabilizing fatigue-effects does not appear to exist, sizeable and potentially dangerous destabilization does occur when exercise intensity exceeds 50% of VO_2_max (Nardone et al., 1997). Besides fatigue of lower limbs (Lundin et al., 1993; Yaggie and McGregor, 2002; Corbeil et al., 2003; Dickin and Doan, 2008; Pinsault and Vuillerme, 2008; Springer and Pincivero, 2009; Paillard et al., 2010; Gimmon et al., 2011; Bisson et al., 2012; Chaubet et al., 2012; Chaubet and Paillard, 2012; Hlavackova and Vuillerme, 2012; Magalhães and Kohn, 2012; Yu et al., 2014; Thiele et al., 2015; Barbieri et al., 2019), trunk (Davidson et al., 2004; Pline et al., 2005; Parreira et al., 2013), neck (Schieppati et al., 2003; Vuillerme et al., 2005; Liang et al., 2014) and whole body (Nardone et al., 1997, 1998; Zemková et al., 2007; Fox et al., 2008; Demura and Uchiyama, 2009; Springer and Pincivero, 2009; Wojciechowska-Maszkowska et al., 2012; Wright et al., 2013; Hill et al., 2014, 2015; Marcolin et al., 2019; Güler et al., 2020), also hyperventilation (Zemková and Hamar, 2003; David et al., 2010, 2015; Malakhov et al., 2014) and/or increased oxygen uptake (Bove et al., 2007) play a main role in post-exercise postural stability. Other possible physiological mechanisms of post-exercise increase in postural sway include impairments of visual cues, vestibular system and proprioceptive functions (Lepers et al., 1997; Hashiba, 1998; Derave et al., 2002; Zemková et al., 2007; Mello et al., 2009), muscle damage (Twist et al., 2008), dehydration (Derave et al., 1998; Lion et al., 2010), hyperthermia (Mtibaa et al., 2018), and dizziness resulting from hyperventilation. Nevertheless, more research is needed to investigate the association of these factors with sport-specific exercises and their effect on athlete performance with high demands on postural stability.

Balance impairment after resistance exercises is also a consequence of more pronounced ventilation rather than fatigue (Zemková and Hamar, 2009a). This effect is more evident after exercises performed with lower (squats and calf rises) than upper extremities (biceps curls and presses behind the neck). The sway velocity after voluntary hyperventilation reaches a maximum at the end of exercise, and starts to decline immediately in the recovery phase. However, its values after resistance exercises, especially after those performed with lower limbs, remain temporarily elevated and a gradual decrease back to the resting level set in only after about 10 and 25 s. This effect is mainly a consequence of the delayed activation of ventilation in an early phase of recovery after such exercises. This assumption may be corroborated by the close correlation between the level of ventilation and sway velocity in the recovery phase (Zemková and Hamar, 2009a). Besides the type of exercise and muscle mass activated, postural sway response to resistance exercises depends on their contraction intensity (additional load used), rate of movement, number of repetitions and sets, and the intensity of proprioceptive stimulation. However, physiological mechanisms underlying changes following resistance exercises performed under sport-specific conditions have yet to be investigated.

These findings have to be taken into account in sports dependent upon post-exercise postural stability, such as biathlon, figure skating, rock and roll dancing, and so forth. Such investigations are essential for designing sport-specific training programs tailored to individual needs. For instance, a recent study by Hill et al. (2016) investigated whether high-intensity cycling training leads to adapted responses of balance performance in response to exercise-induced muscle fatigue. The authors found that 3 weeks of high-intensity training (HIT) on a cycle ergometer resulted in longer recovery times following fatigue compared to pre-training assessments. After 6 weeks of HIT, postural sway following fatigue was attenuated. A better understanding of acute and adaptive changes in postural sway after various exercises may help athletes to improve their performance and decrease the risk of injuries under fatigue conditions.

## Conclusion

This scoping review revealed that among a variety of studies evaluating the effects of exercise on postural balance control, only few of them were tested under sport-specific conditions (i.e., while shooting in biathlon or pentathlon, and after simulated or match-induced protocols in combat and team sports). Therefore, more research is still needed to address this gap in the literature and aim research at the investigation of postural sway response to sport-specific exercises. Further analysis of the existing literature showed that the type, intensity and duration of exercise plays a main role in increased postural sway. Whole body and localized muscular fatigue of the trunk, neck and lower limbs is considered as a main factor responsible for the magnitude of balance impairment in an initial phase of recovery and speed of its readjustment to a pre-exercise level. Hyperventilation and deterioration of sensorimotor functions can also be included, though some contribution of muscle damage, dehydration, hyperthermia or dizziness cannot be excluded. A better understanding of the physiological mechanisms of balance impairment after exercises performed under simulated fatigue induced protocols close to conditions specific to a particular sport, has implications for designing smart exercise programs tailored to individual needs and to improve athlete performance with high demands on postural stability and/or decrease their risk of injuries.

## Author Contributions

The author confirms being the sole contributor of this work and has approved it for publication.

## Conflict of Interest

The author declares that the research was conducted in the absence of any commercial or financial relationships that could be construed as a potential conflict of interest.

## Publisher’s Note

All claims expressed in this article are solely those of the authors and do not necessarily represent those of their affiliated organizations, or those of the publisher, the editors and the reviewers. Any product that may be evaluated in this article, or claim that may be made by its manufacturer, is not guaranteed or endorsed by the publisher.
