# Supplementary material for: Physiological Mechanisms of Exercise and Its Effects on Postural Sway: Does Sport Make a Difference?
Source: Front Physiol. 2022 Feb 14;13:792875. doi: 10.3389/fphys.2022.792875 (PMC8908905; doi:10.3389/fphys.2022.792875)
Supplement: Supplementary file 1 [file Table_1.pdf]

TABLE 1 Postural sway response to various exercises and their underlying physiological mechanisms.

Authors	Study design	Participants	Postural sway response to exercise	Post-exercise balance changes and their physiological mechanisms
Aalto et al. (1990)	A race simulating actual race conditions	10 competition shooters	Shooters have significantly better stability than untrained control subjects when tested without supportive clothing;<br>The competition clothing reduces the sway velocity both in visual and non-visual conditions	The Romberg quotient is higher in shooters than in normal controls, indicating that the shooters use an increased amount of proprioceptive and vestibular cues to stabilize their posture
Seliga et al. (1991)	Light (40 W), moderate (85 W), and heavy (125 W) work loads under conditions of wearing a full facepiece respirator and not wearing any respiratory protection device	15 physically fit males	Sway increases more quickly and in a more consistently linear fashion with increasing work load under the respirator than non-respirator condition	The greater increase in sway during the postural balance test may be attributable to the increasing work load-induced proprioceptive fatigue effect on the nervous system's ability to process signals from proprioception systems incongruent with body sway
Hoffman et al. (1992)	Exercises on the cycle ergometer eliciting heart rates of 130, 150, and 170 beats.min^{-1} and brief maximal exercise	13 biathletes (7 females, 6 males)	The intensity of exertion immediately prior to biathlon shooting has minimal influence on prone shooting performance, but does affect shooting in the standing position by altering the stability of the hold	There is no significant effect of exercise intensity on any measure of shooting accuracy or precision for prone shooting;<br>Measures of shooting precision (group diameter) and shooting accuracy (shot score) are significantly altered by the level of exertion prior to shooting in the standing position
Lundin et al. (1993)	Plantar flexor and dorsiflexor fatigue	8 uninjured male subjects	Significant increase in both medial-lateral and anterior-posterior postural sway amplitude	Fatigue of the plantar flexors and dorsiflexors increases postural sway amplitude and may render the ankle joint susceptible to injury
Adlerton and Moritz (1996)	Fatiguing calf-muscle exercise	13 healthy subjects	Repeated measurements for up to 10 min after exercises don't increase of body sway	Postural control in quiet standing can be maintained by compensatory mechanisms activated during muscle fatigue
Lepers et al. (1997)	25-km run (average time 1 h 44 min) and ergocycle exercise of identical duration	9 well-trained subjects (4 athletes and 5 triathletes)	The ability to maintain postural stability during conflicting sensory conditions decreases after prolonged exercise, with some differences depending on the kind of exercise (running vs. cycling)	Subjects use less effective vestibular inputs after running than after cycling;<br>Adaptation to prolonged stimulation of proprioceptive, vestibular and visual inputs probably occurs in the integrating centres during exercise

Nardone et al. (1997)	Treadmill walking and cycle ergometer pedalling performed under both fatiguing (above anaerobic threshold) and non-fatiguing conditions	13 young subjects	Body sway increases after strenuous physical exercise, but is little affected by exercise performed below the estimated anaerobic threshold	The effect of fatigue on sway is short-lasting and of moderate extent; therefore it is not liable to seriously threaten body equilibrium
Derave et al. (1998)	Cycling for 2 h at a power output equal to 57-63% VO_2max on two different occasions: without drinking and with intake of 1.9 l of a carbohydrate-electrolyte solution	8 male subjects	Mean velocity of CoP excursion after the exercise test is significantly higher in the trial without drinking than with intake of 1.9 l of a carbohydrate-electrolyte solution	Prolonged exercise without fluid ingestion negatively affects postural stability, whereas no effect is observed after exercise with fluid replacement or after thermal dehydration
Hashiba (1998)	Running at 10 km/h and walking at 7 km/h on a treadmill for 7 min.	24 healthy subjects	Mean fore-back postural sway is significantly greater after treadmill than normal running	Vision during treadmill locomotion plays an important role in evoking postural sway after treadmill locomotion;<br>Somatosensory/motor signals may be stored during visual-somatosensory/motor conflict and this stored information may evoke postural change and self-motion perception
Nardone et al. (1998)	Treadmill walking for 25 min.	8 young able-bodied subjects	Body sway is affected by prolonged fatiguing exercise, such as strenuous walking;<br>This effect is of moderate extent and vanishes within a few minutes	A walking exercise, even if strenuous, hardly affects body equilibrium, perhaps because of the redundant mechanisms involved in balance control during upright stance
Adlerton and Moritz (2001)	Muscle fatigue on vibration-perturbed one-leg stance	Healthy young (20-26 years of age) and older (50-60 years of age) women	An immediate but short-lasting effect of fatiguing exercise on vibration-induced CoP oscillations in terms of increased amplitude	Fatiguing exercise does not influence the shift of CoP caused by vibration, thus indicating unchanged excitability of muscle spindles in fatigued muscles
Derave et al. (2002)	30 min of treadmill walking (1.9 m/s) and running (2.2 m/s)	9 healthy subjects	Exercise of moderate intensity deteriorates visual contribution to postural stability;<br>The effect is evident as an initial destabilisation in the sagittal direction and a less transient loss of latero-lateral stability	Running tends to disturb postural stability more than walking, possibly due to more excessive head movement and disturbance of vestibular and visual information centres
Gauchard et al. (2002)	Maximal oxygen uptake (VO_2max) and submaximal exercises in no-hydrated and hydrated conditions at a power corresponding to	10 healthy subjects who regularly practice sports activities	The postural control performance decreases from the best to the worst: control, hydration, dehydration, and VO_2max	Fatigue mainly alters muscular effectors and sensory inputs, such as proprioception, resulting in poor postural regulation;<br>Fluid ingestion may be responsible for the preservation of muscular functions and of

	approximately 60% of the VO_2max of each subject			sensory afferences accurately regulating postural control
Yaggie and McGregor (2002)	Fatigue of the plantarflexors and dorsiflexors induced by isokinetic contractions	24 healthy young men without ankle trauma within 2 years	Isokinetic fatigue of ankle plantarflexors and dorsiflexors significantly influences sway parameters and ranges of postural control;<br>These perturbations are transient and recovery occurs within 20 minutes	Fatigue of ankle plantarflexors and dorsiflexors
Corbeil et al. (2003)	Repeated plantar-flexion of both legs	11 healthy male subjects	Fatigue of ankle plantar-flexors places higher demands on the postural control system by increasing the frequency of actions needed to regulate the upright stance	Fatigue induces some changes in the control mode of postural stability, but the detection/action capabilities of the sensorimotor system remains partly efficient when the ankle plantar-flexors are fatigued;<br>The decreased long-term scaling exponent observed with fatigue suggests that the control of upright stance operates in a less stochastic and more antipersistent manner when fatigue is present (i.e. past and future behaviors are more negatively correlated and thus more tightly regulated)
Davidson et al. (2004)	A protocol that fatigued the lumbar extensors to 60% of their unfatigued maximum voluntary exertion force;<br>The rate of fatigue (high vs low) controlled by the duration of fatiguing exercises (10 min vs 90 min)	13 physically active young males	An increase up to 58% in time-domain postural sway measures, but no change in frequency-domain measures;<br>Fatigue rate does not affect the magnitude of postural sway increases, nor does it affect the rate of balance recovery following fatigue	There is no significance between fatigue rates, but subjects recover more quickly after the high fatigue rate protocol
Pline et al. (2005)	Ankle joint motion sense evaluated with and without circumferential ankle pressure before and after the lumbar extensors were fatigued	14 healthy male subjects	Muscle fatigue of the lumbar extensors decreases ankle joint motion sense;<br>The application of circumferential ankle pressure decreases ankle joint motion sense	Lumbar extensor fatigue impairs ankle proprioceptive acuity, which may explain increases in postural sway subsequent to lumbar extensor fatigue
Vuillerme et al. (2005)	No fatigue and fatigue of the scapula elevator muscles	14 young healthy adults	The cervical muscular fatigue increases CoP displacements in the absence of vision;<br>This effect is more accentuated when somatosensation is degraded by standing on a foam surface;<br>The availability of vision allows the individuals to suppress this destabilising effect	The importance of intact cervical neuromuscular function on postural control during quiet standing;<br>A reweigthing of sensory cues in balance control following cervical muscular fatigue by increasing the reliance on the somatosensory inputs from the plantar soles and the ankles and visual information

Bove et al. (2007)	Strenuous treadmill exercise and a 3s-bilateral soleus muscle vibration after the strenuous exercise	20 healthy young subjects (15 males, 5 females)	There is a linear relationship between sway path and oxygen uptake	The short duration of body instability may be due to the quite rapid recovery of oxygen uptake;<br>The fatigue-induced body instability does not associate with changes in the early postural response to soleus muscle vibration
Strang and Berg (2007)	A dead-lift exercise performed to exhaustion	30 healthy young adults	Fatigue has no effect on postural stability during the focal movement, and yet causes earlier anticipatory postural adjustment (APA) onsets in the contralateral paraspinals, ipsilateral paraspinals and contralateral paraspinals	Early APA onset may enhance postural stability by permitting a longer duration APA, which can counteract fatigue-induced decreases in the force-producing capability of muscles that contribute to postural stability
Dickin and Doan (2008)	Isokinetic concentric actions of the flexors and extensors of the ankle or knee joint;<br>Repeated squat jumps, involving both eccentric and concentric actions to induce fatigue in the whole lower extremity	16 healthy college-aged individuals (9 males, 7 females)	Muscular fatigue imposes a prolonged internal perturbation to postural control, regardless of any individual or combined joint fatigue localization	This global effect, combined with the prolonged impairment in postural response, provides support for critical contributions from a central mechanism to postural deficits due to fatigue
Fox et al. (2008)	Anaerobic (maximum-effort sprints) and aerobic (yo-yo intermittent recovery test, level 1) exercise protocols	36 collegiate athletes	Postural control is negatively affected after anaerobic and aerobic exercise protocols as measured by total Balance Error Scoring System score, elliptical sway area, and sway velocity	The effects of fatigue persists for up to 13 minutes before postural control returns to baseline
Pinsault and Vuillerme (2008)	No fatigue and fatigue of the ankle plantar-flexor muscles induced by toe-lifts performed until exhaustion	30 male university students	The fatigue condition increases CoP displacements relative to the no fatigue condition;<br>This destabilizing effect is more accentuated in the head tilted-backward posture than neutral head posture;<br>This destabilizing effect is less accentuated in the condition of tactile stimulation than that of no tactile stimulation of the neck	There is an increased reliance on vestibular and neck somatosensory information for controlling posture during quiet standing in condition of altered ankle neuromuscular function
Twist et al. (2008)	Plyometric exercise consisting of 200 countermovement jumps designed to elicit symptoms of muscle damage	9 healthy adults	There is a latent impairment of balance performance following a bout of plyometric exercise;<br>The stability index significantly increases above baseline	Perceived muscle soreness of the calf region increases significantly following the plyometric exercise protocol

Bizid et al. (2009)	Fatiguing of the triceps surae, either by voluntary muscular contractions (group VOL) or by electrical stimulation superimposed onto voluntary muscle contractions (group VOL+ES)	16 healthy male subjects	Calf-muscles fatigue induces a modification of the postural strategy that may be exerted more strongly after voluntary muscular contractions than after electrical stimulation superimposed onto voluntary muscular contractions	Calf-muscles fatigue does not impair postural control but it generates a greater change of the contribution of the proprioceptive information (myotatic loops) in group VOL than in group VOL+ES
Demura and Uchiyama (2009)	Anaerobic exercise: two times of a maximal voluntary pedaling for 10 seconds;<br>Aerobic exercise: pedaling at 50% of maximal aerobic power for 60 minutes at 60 rpm	15 healthy male subjects	CoP sway area and velocity are significantly higher immediately after both exercises	Both prolonged aerobic exercise and high-intensity anaerobic exercise have a relatively small influence on upright standing postural control in healthy young males;<br>The exercise-induced increase of sway velocity recovers earlier than the physiological parameters (heart rate, systolic blood pressure, and blood lactate concentration)
Mello et al. (2009)	The maximal oxygen uptake test and prolonged cycle ergometer exercise	16 healthy male subjects	The changes in postural control are dependent on the intensity and duration of exercise;<br>The maximal oxygen uptake test decreases the mean duration of peaks in sway density plot (SDP), thus decreasing the stability level, without modifying the rates of central and muscular torque controls;<br>A 60 min exercise increases the mean time interval between two consecutive peaks in SDP, thus decreasing the control rate but not changing the stability level	Visual privation has a greater effect on body sway than exercises, which are applied to muscles that are not the main actuators in body sway control
Springer and Pincivero (2009)	Localized muscle and whole-body fatigue consisting of single-leg, weight-bearing heel raises on an inclined platform, and exercise on a rowing ergometer, respectively, to the point of volitional failure	20 healthy young subjects (10 males, 10 females)	The localized muscle and whole-body fatigue increases medial/lateral and total sway;<br>Total sway variability increases significantly more following the localized muscle fatigue protocol than the whole-body fatigue protocol	Whole-body fatiguing exercise, while maintaining a non-active state of the controlling lower limb muscles, is just as detrimental as isolated muscle fatigue
Vuillerme et al. (2009)	Fatiguing exercise for the hip abductors of their dominant leg	24 young healthy adults	Unilateral muscle fatigue induced on the hip's abductors of the dominant leg has different effects on the plantar CoP displacements under the non-fatigued and	Postural responses could be viewed as an adaptive process to cope with an unilateral alteration in the hip neuromuscular

			fatigued legs, i.e. yielding larger displacements under the non-fatigued leg only, and in the anteroposterior and mediolateral axes, yielding larger displacements along the mediolateral axis only	function induced by the fatiguing exercise for controlling bipedal stance;<br>The increase in CoP displacements under the non-fatigued leg in the fatigue condition could reflect enhanced exploratory "testing of the ground" movements with sensors of the non-fatigued leg's feet, providing supplementary somatosensory inputs to the central nervous system to preserve/facilitate postural control in condition of altered neuromuscular function of the dominant leg's hip abductors induced by the fatiguing exercise
Zemková and Hamar (2009b)	Soccer match	10 soccer players	CoP velocity under dynamic conditions with eyes closed increases after the first half of the game, whereas with eyes open increases after the second half of the game;<br>There are no significant changes in static balance with eyes open and eyes closed	Soccer match induced fatigue increases the drop jump ground contact time, concomitant with the impairment of dynamic balance and agility performance when moving over a short distance;<br>There are no changes in the agility performance over a longer movement distance, explosive power of lower limbs, speed of step initiation, speed of the soccer kick, and static balance
David et al. (2010)	Maximum-intensity, incremental cycling exercise test	12 healthy subjects	Hyperventilation induced by exercise is accompanied by a significant increase in postural parameters, indicating a reduction in postural stability following a change in ventilatory drive;<br>Coherence analysis confirms the ventilatory origin of postural oscillations	Ventilation may be an important factor in postural disturbance during physical activity;<br>The increases in leg muscle activities are most likely related to musculo-articular stiffening
Lion et al. (2010)	45 min. exercise on a cycle ergometer at intensity just below the ventilatory threshold without fluid intake	10 sportsmen	Postural performances decreases immediately after exercise, mainly in the standard situation (eyes open, stable visual surround and platform) and when only the vestibular cue is reliable (eyes closed and sway-referenced platform)	Even though muscular fatigue could explain the decrease in postural performances, vestibular fluid modifications may also be involved by its influence on the intralabyrinthine homeostasis, lowering thus the contribution of vestibular information on balance control

Paillard et al. (2010)	The fatigue of the quadriceps femoris induced by voluntary muscular contraction (VC), and by electrical stimulation superimposed onto voluntary muscular contraction (VC+ES)	14 healthy young adults	Superimposing electrical stimulation onto VCs impaires muscle strength and postural control less than do VCs alone;<br>The duration of recovery of these two neurophysiological functions does not differ for the two fatiguing exercises;<br>For both exercises, postural control is restored faster than the ability to produce muscular strength	Electrical stimulation superimposed onto voluntary contraction may limit the deterioration of muscular fatigue and postural control
Boyas et al. (2011)	Isokinetic fatigue task involving either only plantarflexors (PFs) (30°s^{-1}), only dorsiflexors (DFs) (120°s^{-1}), or both PFs and DFs simultaneously;<br>The fatigue task involved maximal contractions repeated until the torque produced decreased below 50% of the maximal torque	16 adults (8 women, 8 men)	There is no effect of fatigue on postural variables with eyes open;<br>Sway area and anteroposterior velocity with eyes closed increases only when both PFs and DFs are fatigued simultaneously	An effect of fatigue present only when both muscle groups are fatigued simultaneously could be due to impairment in the compensatory activity between agonist and antagonist muscles and/or a greater decrease in proprioception due to a greater number of fatigued muscles
Gimmon et al. (2011)	Non-fatigue standing on firm surface; non-fatigue standing on foam; ankle plantar flexor fatigue, standing on firm surface; ankle plantar flexor fatigue, standing on foam; upper limb fatigue, standing on firm surface	10 healthy young volunteers	Localized plantar flexor fatigue causes impairment of postural control mainly in the sagittal plane;<br>Traditional CoP parameters are affected by plantar flexor fatigue, especially in the anteroposterior direction;<br>For the SDA parameters, plantar flexor fatigue causes significantly higher short-term diffusion coefficients, and critical displacement in both mediolateral and anteroposterior directions;<br>Long-term postural sway is different only in the anteroposterior direction	Postural corrections occur at a higher threshold of sway during plantar flexor fatigue compared to non-fatigue conditions
Bisson et al. (2012)	An isometric and an isokinetic fatiguing exercise	10 healthy young men	Both fatiguing exercises increase CoP excursion area, CoP variability and CoP velocity in both planes (anteroposterior, mediolateral) on the compliant surface;<br>Both fatiguing exercises increase CoP variability and CoP velocity in the fatigued plane (anteroposterior) on the firm surface	The effects of fatigue on postural control are more pronounced when standing on a compliant surface, i.e. when proprioceptive information at the ankle is altered

Chaubet et al. (2012)	Fatigue of the quadriceps femoris after fatiguing voluntary contractions (VOL) and fatiguing neuromuscular electrical stimulation (ES)	19 active male subjects	The ES exercise affects MVC more than the VOL exercise;<br>Bipedal postural control is similarly deteriorated for both exercises;<br>For both fatiguing exercises, muscle strength and postural control does not recover their initial level after a 5 min recovery	The postural control disturbance could not be distinguished for the two fatiguing exercises in the bipedal stance;<br>The recovery speeds of postural control and muscle strength abilities does not differ for the ES exercise and the VOL exercise
Chaubet and Paillard (2012)	Unilateral knee extensor fatigue induced by electrically stimulated (ES) and voluntary (VOL) contractions	17 healthy active males	Unilateral knee muscle fatigue induced by ES similarly degrades the bipedal postural control as that induced by VOL;<br>The duration of the recovery of postural control does not differ between both fatiguing exercises	The disturbance of the bipedal postural control after unilateral knee muscle fatigue is not only related to a reduction in muscle strength but also to an impairment of the effectiveness of sensory inputs
Hlavackova and Vuillerme (2012)	Muscle fatigue induced in the plantar-flexor muscles of both legs through the execution of a repeated standing heel raise exercise	22 young healthy adults	Plantar-flexor muscle fatigue increases centre of foot pressure displacements under normal foot and ankle sensory conditions;<br>This effect is exacerbated under altered foot and ankle sensory conditions and mitigated under improved foot and ankle sensory conditions	There is an increased reliance on somatosensory information from the foot and ankle for controlling upright posture in the presence of plantar-flexor muscle fatigue
Magalhães and Kohn (2012)	Subsensory electrical noise stimulation applied over the triceps surae (TS)	11 healthy physically active subjects (9 males, 2 females)	There is a significant correlation between the reduction in force fluctuation and the decrease in postural sway with the electrical noise stimulation	Changes in plantar flexion force variability in response to a given subsensory random stimulation of the TS may provide an estimate of the variations in postural sway caused by the same subsensory stimulation of the TS;<br>Decreases in force variability and postural sway are due to stochastic resonance that causes an improved transmission of proprioceptive information
Mel'nikov et al. (2012)	PWC(170) cycle test	31 sambo wrestlers	Linear sway velocity after PWC(170) in bipedal stance increases to an equal extent in wrestlers and controls but the increase of CoP velocities in squat position are lower in athletes and are negatively correlated to an extent of recovery of heart rate after PWC(170), indicating some contribution of an recovery rate to maintenance of a high level of postural stability in a static strain position in wrestlers during physical fatigue	Parameters of sway increase in both bipedal stance and squat position after PWC(170) test indicating an important role of muscular fatigue in the decrease of postural stability

Wojciechowska-Maszkowska et al. (2012)	Anaerobic Wingate test on a cycle ergometer	12 taekwondo practitioners	Intensive physical exercise has an adverse effect on dynamics of body control;<br>The impairment is more pronounced in sagittal plane	Eye sight is an important factor of stable body posture control in the state of fatigue
Zech et al. (2012)	Whole-body and localized fatigue induced by treadmill running and single-leg step-up exercises	19 male handball players	The CoP sway velocity increases significantly after general and localized fatigue;<br>There is no fatigue effects for the star excursion balance test (SEBT);<br>There are no significant correlations between CoP sway velocity and SEBT mean reach in any condition	Although fatigue affects static postural control, sensorimotor mechanisms responsible for regaining dynamic balance in healthy athletes seem to remain predominantly intact
Boyas et al. (2013)	Fatiguing exercises consisting of sustaining plantarflexor isometric contractions at different intensities of 25, 50 and 75% of maximal isometric plantarflexor torque until task failure	18 young subjects	All fatiguing exercises induce similar alterations in postural stability;<br>Fatiguing exercise intensity does not influence the extent of postural stability impairment, but does influence the type of fatigue induced and the neuromuscular function predictors explaining changes in postural variables	Fatigue-related changes in postural stability parameters are related to neuromuscular function and could explain more than half of the changes in postural variables for the 25% exercise, whereas are less predictive for the 50 and 75% exercises
Parreira et al. (2013)	Fatiguing trunk extension-flexion exercise on a roman chair carried out to exhaustion	18 young and 18 elderly adults	There is an increase in sway immediately post-fatigue compared with pre-fatigue values for all CoP parameters in both groups;<br>The differences are significant only for the CoP velocity parameter, with more pronounced effects in young adults that do not return to pre-fatigue values at the end of 20 min recovery	There is a significant effect of trunk extensor muscle fatigue on postural control, which is more evident in young adults than in the elderly
Steib et al. (2013)	Fatiguing treadmill running	30 active athletes (14 with a previous severe ankle sprain and 16 uninjured controls)	Fatiguing running significantly affects static and dynamic postural control in participants with a history of ankle sprain;<br>Fatigue-induced alterations of dynamic postural control are greater in athletes with a previous ankle sprain	Even after successful return to competition, ongoing deficits in sensorimotor control may contribute to the enhanced ankle reinjury risk
Wright et al. (2013)	Incremental tests on a treadmill and a cycle ergometer	16 recreationally trained individuals (11 male, 5 female)	Balance increases significantly in the overall stability index (SI) and the anterior/posterior index (API) immediately following the fatiguing treadmill test;<br>Balance is not altered significantly in SI or API immediately following the fatiguing cycle test;	Effects of fatigue on balance are seen immediately and are diminished as time after exercise increases

			Balance is not altered significantly for the medial/lateral index for either exercise test at any time point;<br>There are no significant differences in time to recovery, all indices are below pre-exercise values at 12-min post-exercise	
Hill et al. (2014)	Five separate exercise trials consisting of two incremental exercise tests to exhaustion for arm crank ergometry (ACE) and cycle ergometry (CE), two tests of 30 min duration for ACE and CE at a relative workload corresponding to 50% of the ergometer-specific Wmax, CE trial at the same absolute power output as the submaximal ACE trial to match absolute exercise intensity	9 healthy, non-specifically trained adults	ACE has no effects on postural sway;<br>There is an increase in mediolateral CoP displacement following maximal CE only, while anteroposterior CoP displacement and CoP path length increase following maximal and submaximal CE	Differences in postural sway according to exercise mode likely stem from the activity of postural muscles when considering that CE recruits lower limb muscles involved in balance
Liang et al. (2014)	Repeated weight-resisted scapular elevation exercises	48 young healthy adults	Traditional measures of CoP velocity and standard deviation increase monotonically with neck muscle fatigue or without vision;<br>CoP velocity within the ultralow and moderate frequency bands increases post-fatigue;<br>CoP velocity increases in all but the ultralow frequency band without vision	Post-fatigue, vision may be the main compensatory postural mechanism for altered neck proprioception
Yu et al. (2014)	Transcutaneous electrical nerve stimulation (TENS) on fatigued dorsi-plantar flexor with and without visual input	13 healthy adult males and 12 females	When compared of dorsi-plantar flexor after applied to TENS without visual input, the postural sway of plantar flexor is lower than the dorsi flexor	The application of TENS in GCM clinically decreases the postural sway and with visual input it helps to stable posture control and prevent to falling down
Clarke et al. (2015)	Fatiguing game simulation (G-Sim)	15 male collegiate football players	There is a larger area of the CoP trajectory during a 60-s postural sway task after a Canadian football G-Sim	Acute neuromuscular impairment is likely attributable to alterations in excitation-contraction coupling due to structural damage and central activation failure;<br>Congruency between the direct and indirect measures of neuromuscular fatigue suggests that monitoring postural sway has the potential to identify both

				neuromuscular and somatosensory alterations induced by acute game-induced fatigue in collision-based team sports players
David et al. (2015)	Maximum-intensity, incremental cycling exercise test	12 healthy subjects	The change in ventilatory drive induced by exercise is accompanied by a significant increase in both postural sway parameters and EMG activities;<br>For obliquus externus and obliquus internus, the increased EMG activities are prominent during expiration, whereas obliquus internus is silent during inspiration;<br>Obliquus externus and rectus abdominis are activated during both expiration and inspiration	The compensation of respiratory disturbances of the erect posture appears to be less effective when minute ventilation increases;<br>The patterns of muscle activity suggest that abdominal muscles are controlled differentially and that their functional coordination is dependent on the respiratory demand
Hill et al. (2015)	Three incremental exercise tests to 85% of individual's maximal heart rate for arm crank ergometry (ACE), cycle ergometry (CE) and treadmill walking (TM);<br>Subsequent tests involving 20-min of ACE, CE and TM exercise at a relative workload corresponding to 50% of each individual's predetermined heart rate reserve	9 healthy older females	CE and TM elicite significant post exercise balance impairments, which last for ∼10 min post exercise;<br>Postural stability is not impaired after ACE	Quiet standing balance is affected following CE and TM, but lasts only 5–10 min;<br>ACE performed at the same relative intensity as CE and TM do not elicit increase in postural sway
Thiele et al. (2015)	Five sets of eight repetitions of free-weight back-squat exercises at 80% of one-repetition maximum	18 college-aged, resistance-trained males	Sway index significantly increases following completion of the back-squat, however it recovers within 5 min of exercise	Higher sway index values are a result of neuromuscular fatigue induced by a back-squat exercise;<br>Balance impairments may recover in ~5 min following high-intensity lower body resistance exercise
Barbieri et al. (2019)	(1) Saccadic eye movements;<br>(2) Maximum voluntary isometric contractions in a leg press device, custom-made to test ankle plantar flexion force;	20 young male adults	Body sway increases after induction of ankle muscle fatigue;<br>Saccadic eye movements consistently reduce postural sway in fatigued and unfatigued conditions;<br>Closing the eyes increases sway in the unfatigued condition but reduces sway in the fatigued condition	Individuals can adjust sensory weights to improve postural control after ankle muscle fatigue, but these adjustments are dependent upon vision conditions;<br>Eye movement or vision manipulations may serve as a strategy for increasing postural stability under ankle muscle fatigue

	(3) A calf raise exercise on top of a step to induce ankle muscle fatigue;<br>(4) A repetition of items 1 and 2			
Marcolin et al. (2019)	25 min of moderate running on a treadmill	8 female and 6 male healthy subjects	The area of the confident ellipse, sway path velocity, sway area velocity, and medio-lateral maximal oscillation significantly increases after the treadmill run;<br>There are no significant differences in both short and long term diffusion coefficients;<br>There are no significant differences in the parameters of the dynamic postural stability test	There is a dissimilar trend of static (worsening) and dynamic (not statistically significant improvement) postural stability after physical exercise
Sadowska et al. (2019a)	Maximal specific physical effort performed on a ski ergometer and continued until exhaustion	10 junior elite biathletes	Maximal physical effort exerts a significant effect on postural sway and rifle sway characteristics;<br>There is higher post-effort rifle sway in the vertical direction than in the across the shooting line direction;<br>Post-effort postural balance impairment in the shooting line is much greater than in the across the shooting line direction;<br>There is a strong correlation between postural balance and rifle stability	Rifle sway during aiming in a standing shooting position seems to be coordinated with the postural sway of the biathlete's body
Sadowska et al. (2019b)	Laser Run combined event as a part of the modern pentathlon, consisting of successive shooting and running phases	25 modern pentathletes	There are significant changes in the average velocity of the CoP and the width of the ellipse of the CoP shifts area between the first and the second shooting series	The fatigue caused by the running phases in the Laser Run affects the stability of the shooting position of pentathletes;<br>Disturbances that occur after the first running phase are maintained at the same level during the subsequent shooting series;<br>The fatigue level does not affect the magnitude of the disturbances of the postural balance in the shooting position
Güler et al. (2020)	Anaerobic exercise: four maximal cycling efforts against a resistance equivalent to 0.075 kg/body mass for 30 s with 3 min rest intervals;	16 female soccer players	There is higher impairment of balance after the aerobic than anaerobic loading;<br>The time-related deterioration after both aerobic and anaerobic loadings is similar	The fatigue induced by aerobic and anaerobic exercise negatively affects a single-leg dynamic balance;<br>Single leg balance ability returns to the baseline after 10 min of passive recovery

	Aerobic exercise: the Bruce protocol on a motorized treadmill			
Lyu et al. (2021)	Local fatiguing exercise: intermittent isometric knee extensions at the level of 40% of maximal voluntary torques; General fatiguing exercise: rowing ergometry at a speed of 200 ± 5 m/min	7 young male subjects	Disturbed postural tests: no significant change of anticipatory postural adjustment organizations in individual muscles following both fatiguing exercises, but observed larger anticipatory postural adjustment coactivations in trunk and dorsal muscle pairs following local than general fatiguing exercise, and larger compensatory postural adjustments coactivation in dorsal muscle pair after both fatiguing exercises; Static postural tests: efficient static postural stability accompanying the down-weighting of visual input and the up-weighting of vestibular/somatosensory component following both fatiguing exercises	There is a general compensation in the central nervous system in response to the neuromuscular deficiencies induced by local fatiguing exercise which put forward the function of sensory recalibration in maintaining postural stability under fatigue conditions
